# Comprehensive Analysis of the Expression and Prognosis for the DREAM Complex in Human Cancers

**DOI:** 10.3389/fgene.2022.814725

**Published:** 2022-05-12

**Authors:** Lulu Wang, Xiaowei Liu

**Affiliations:** ^1^ Yangpu Hospital, School of Medicine, Tongji University, Shanghai, China; ^2^ Department of Pediatric Surgery, Xinhua Hospital, School of Medicine, Shanghai Jiaotong University, Shanghai, China; ^3^ Division of Pediatric Oncology, Shanghai Institute of Pediatric Research, Shanghai, China

**Keywords:** DREAM complex, genomics, methylation, pan-cancer, prognosis

## Abstract

The DREAM complex is an evolutionarily conserved cell cycle regulating multi-protein complex. In addition to playing an essential function in the cell cycle, it also plays a vital role in various survival activities. Accumulating evidence suggests that the DREAM complex plays a crucial role in oncogenesis. However, the regulatory mechanism of the DREAM complex in cancer remains unclear. This study used multi-omics data from Cancer Genome Atlas and Cancer Cell Line Encyclopedia to comprehensively identify the DREAM complex in tumor samples from 33 cancer types. In the genomic landscape, we identified the missense mutation as the dominant alteration events. Expression analysis showed that the expression of methylation-mediated the DREAM complex was downregulated. In addition, we found that the expression of the DREAM complex can be performed to predict the survival of various cancer patients. Pathway activation analysis showed that the DREAM complex is related to apoptosis inhibition, cell cycle, DNA damage response, RAS/MAPK, and RTK signaling pathway activation. Importantly, through a comprehensive analysis of drug sensitivity genomics in cancer databases, we identified a number of potential drugs that may target the DREAM complex. In summary, this study revealed the genomic changes and clinical features of the DREAM complex in 33 cancers, which may also provide new insights for cancer treatment and may offer alternative options for the treatment of clinically refractory cancers.

## Introduction

The DREAM complex, also named the LINC complex, is an evolutionarily conserved cell cycle regulation polyprotein complex (Lewis et al., 2004; Harrison et al., 2006; Litovchick et al., 2007; Schmit et al., 2007). The DREAM complex, associated with p130 (retinoblastoma-like protein 2/RBL2), E2F4, and DP1 (E2F dimerization partner 1), exists on the promoters of about 1,000 cell cycle-related genes, inhibiting their transcription and inducing cell silencing (Litovchick et al., 2007; Sadasivam and DeCaprio, 2013; Gal et al., 2021). The DREAM complex including a retinoblastoma family member, an E2F transcription factor and its dimerization partner, and five proteins related to products of MuvB genes LIN-9, LIN -37, LIN -52, LIN -53, and LIN -54 (comprising the MuvB core) have been described in distinct organisms (Pilkinton et al., 2007; Sadasivam and DeCaprio, 2013; Walston et al., 2021). In different stages of the cell cycle, the composition of the core complex does not change. Still, it does interact with various proteins to change its function from transcription repressors of G0 and G1 to activators of S, G2, and M. In the G0 phase and the early G1 phase, the MuvB core interacts with E2F4/5, DP1/2, and RBL2 to inhibit the G1/S and G2/M genes to form the inhibitor DREAM. In the late stage of G1, DREAM-specific protein separates from the MuvB core and then binds to B-MYB in the S phase, which transactivates cell-cycle genes related to the S/G2/M phase (Korenjak et al., 2004; Litovchick et al., 2007; Sadasivam et al., 2012; Fischer et al., 2016).

The DREAM complex plays a vital role in orchestrating the cell cycle, deregulation of this complex is connected with several cancers (Sadasivam and DeCaprio, 2013; MacDonald et al., 2017). However, further research is still needed to elucidate the genomic and clinical characteristics of the DREAM complex in cancer.

In this study, we systematically assessed the genomics and clinical characteristics of the DREAM complex in 33 solid tumors. In addition, we studied the impact of these gene expression changes on clinical outcomes and drug sensitivity. We also explored possible downstream signaling pathways, which the DREAM complex may mediate.

## Materials and Methods

### Dataset and Tumor Types

The Genotype-Tissue Expression (GTEx) dataset (V8.0) (https://commonfund.nih.gov/GTEx/) was used for gene expression analysis of normal tissues. GSCALite (http://bioinfo.life.hust.edu.cn/web/GSCALite/) is an integrated genomic, and immunogenomic web-based platform for gene set cancer research (Liu et al., 2018). Oncomine database (https://www.oncomine.org/resource/login.html, an online cancer microarray database) was referred to examine the expression levels of the DREAM complex mRNA in distinct types of cancers. The baseline expression of the DREAM complex was measured in 30 normal organs/tissues, including adipose tissue, adrenal gland, bladder, blood, blood vessel, brain, breast, cervix uteri, colon, esophagus, fallopian tube, heart, kidney, liver, lung, muscle, nerve, ovary, pancreas, pituitary, salivary gland, skin, small intestine, spleen, stomach, testis, thyroid, uterus, and vagina. Gene expression values were calculated as the Transcripts Per Million (TPM). The cancer proteome atlas (TCPA) (https://tcpaportal.org/tcpa/index.html) database was used to obtain clinical data, single nucleotide variation (SNV) data, copy number variation (CNV) data, and methylation data. The correlation between gene expression and drug sensitivity was collected from The Genomics of Drug Sensitivity in Cancer (GDSC) database (www.cancerrxgene.org). Finally, samples of 33 types of cancer were included into the pan-cancer analysis, as follows: acute myeloid leukemia (LAML), adrenocortical carcinoma (ACC), bladder urothelial carcinoma (BLCA), breast invasive carcinoma (BRCA), cervical squamous cell carcinoma and endocervical adenocarcinoma (CESC), cholangiocarcinoma (CHOL), colon adenocarcinoma (COAD), esophageal carcinoma (ESCA), glioblastoma multiforme (GBM), head and neck squamous cell carcinoma (HNSC), kidney chromophobe (KICH), kidney renal clear cell carcinoma (KIRC), kidney renal papillary cell carcinoma (KIRP), lower grade glioma (LGG), liver hepatocellular carcinoma (LIHC), lung adenocarcinoma (LUAD), lung squamous cell carcinoma (LUSC), lymphoid neoplasm diffuse large B-cell lymphoma (DLBC), mesothelioma (MESO), ovarian serous cystadenocarcinoma (O.V.), pancreatic adenocarcinoma (PAAD), pheochromocytoma and paraganglioma (PCPG), prostate adenocarcinoma (PRAD), rectum adenocarcinoma (READ), sarcoma (SARC), skin cutaneous melanoma (SKCM), stomach adenocarcinoma (STAD), testicular germ cell tumors (TGCT), thymoma (THYM), thyroid carcinoma (THCA), uterine carcinosarcoma (UCS), uterine corpus endometrial carcinoma (UCEC), and uveal melanoma (UVM).

### mRNA Differential Expression Analysis

mRNA Seq data and clinical data were collected from the TCGA database. In the mRNA differential expression analysis, normalized mRNA expression data based on RNA-seq [RNA-Seq V2 RSEM (RNA-Seq by Expectation-Maximization)] was obtained from the TCGA data portal. The number of samples for each cancer type ranged from 48 to 1,098. We performed the analyses across 14 cancer types with sufficient normal tissues (at least 10 samples with matching tumor adjacent normal tissue), including BLCA, BRCA, COAD, ESCA, HNSC, KICH, KIRC, KIRP, LIHC, LUAD, LUSC, PRAD, STAD, and THCA. The RSEM normalized mRNA expression values were used. Fold change is expressed by mean (Tumor)/mean (Normal); the *p*-value was determined using a *t*-test and was adjusted by the false discovery rate (FDR). Genes with fold change (F.C.) >2 and false discovery rate (FDR)-adjusted *p*-value < 0.05 were retained for further analysis. If there was no significant gene in a cancer type, then that cancer type will be excluded from the final analysis.

### Survival Analysis

The mRNA expression data of the DREAM complex in 33 cancers were combined with the corresponding clinical survival data for expression survival analysis. The mRNA expression data of the DREAM complex in 33 cancers were combined with the related clinical survival data for expression survival analysis. According to the median RSEM value, the tumor specimens were divided into high group and low group. Then, we used the R package “survival” to estimate the two groups’ survival time and survival status. The log-rank test was performed in a univariate Cox regression analysis using a proportional hazards model to examine the association between each gene and patient survival separately, and the determined log-rank *p*-values were adjusted after the Benjaminii-Hochberg FDR. Genes with adjusted log-rank *p*-values (also known as Q-values) <= 0.05 are preserved. (Nguyen and Le, 2020).

### Subtype Analysis

Genes may have different expression levels in cancer subtypes. Therefore, we performed the analysis of gene expression in tumor subtypes. We analyzed subtypes in the following cancers: BRCA, KIRC, LUAD, STAD, HNSC, LUSC, BLCA, the number of subgroup of subtype must have at least 10 samples. The method used for clinical correlation analysis depends on the number of subgroups of the subtype: *t*-test and ANOVA *t*-test. The final results included genes and cancer types whose *p*-values were less than 0.05 after statistical testing.

### Single Nucleotide Variation Analysis

We collected TCGA SNV data and evaluated the frequency and clinical effect of several variant types of effective mutations. The TCGA SNV data include variant-type values: Missense_Mutation, Silent, 5′ Flank, 3′ UTR, RNA, In_Frame_Del, Nonsense_Mutation, Splice_Site, Intron, 5′ UTR, In_Frame_Ins, Frame_Shift_ Del, Nonstop_Mutation, 3′ Flank, Frame_Shift_Ins, and Translation_Start_Site. We only considered the SNV of each gene coding region and filtered out silent, Intron, IGR, 3′UTR, 5′UTR, 3′Flank, and 5′Flank SNVs. The calculation formula of SNV mutation frequency (percentage) of each gene coding region is the number of mutation samples/number of cancer samples. An SNV oncoplot plot was generated using maftools (Mayakonda et al., 2018).

### Copy Number Variation Analysis

The raw data of CNVs of 33 cancer types were obtained from the TCGA database and processed using GISTICS2.0 (Mermel et al., 2011). In this CNV module, we calculated the percentage of CNVs in each cancer type, the correlation of CNVs with gene mRNAs. CNV can be divided into two subtypes, namely heterozygous and homozygous, that is, CNV occurs on only one chromosome or on both chromosomes at the same time. The CNV data processed by GISTIC was used for percentage statistics based on CNV subtypes, and the correlation was calculated using the original CNV data and mRNA RSEM data. We only discussed the genes with 5% CNV in cancer. According to the method employed by Schlattl et al. (2011), the mRNA expression and CNV raw data were merged according to their TCGA barcode. We tested the association between paired mRNA expression and paired CNV percent samples on Person’s product-moment correlation coefficient, followed by a T distribution. The FDR adjusted the *p*-value.

### Methylation Analysis

Methylation data were available in the TCGA database. Only 14 cancer types had matching tumor-adjacent normal data. Therefore, differential methylation analysis was based on these 14 cancer types. Cancer types that contain at least 10 paired TCGA normal samples were selected to be calculated, but only paired samples were incorporated. The methylation difference between tumor and normal samples was identified using Student’s t-test at an FDR-adjusted *p*-value <= 0.05. In theory, methylation can lead to the abnormal expression of a gene. We matched methylation and mRNA expression data by patient header I.D. using the TCGA barcode. We further collected the genes whose gene methylation was significantly associated with expression, combined the methylation data with clinical overall survival data, and divided the genes’ methylation level into two groups based on the middle methylation level. A high methylation risk rate was defined as high risk. Otherwise, it was described as low risk. Differences between groups were measured using a log-rank test, and two-tailed *p*-values of less than 0.05 were considered statistically significant.

### Pathway Activity Analysis

Based on the reversed-phase protein array (RPPA) data in the TCPA database, the activity of the downstream signaling pathway was analyzed. According to the method described by Rehan et al. (Akbani et al., 2014). Pathway activity score (PAS) was estimated by the approach adopted by Akbani et al. and Ye et al. (2018). According to the median expression level, the gene expression level was divided into high and Low. The Student’s test determined the PAS difference between the groups. The *p*-value was adjusted for the FDR, and *p* < 0.05 was considered to indicate significant enrichment. When PAS (A high gene group)>PAS (A low gene group), we assessed gene A might have an activating effect on this pathway; otherwise, it had an inhibitory effect on this pathway.

### Drug Sensitivity Analysis

The small molecules were obtained from The Genomics of Drug Sensitivity in Cancer (GDSC) database and The Cancer Therapeutics Response Portal (CTRP). According to Rees et al., we analyzed the correlation between gene expression and drug sensitivity (Basu et al., 2013; Seashore-Ludlow et al., 2015; Rees et al., 2016). To explore the correlation of the DREAM complex and drug sensitivity, the Pearson correlation coefficients of transcript levels and AUCs were used and normalized based on Fisher’s Z transformation. Gene set resistance analysis was performed on GDSC IC50 drug data. Spearman correlation coefficient is used to indicate whether gene expression is related to drug sensitivity.

### Statistical Analysis

The Student’s t-test was used to compare the differences in the DREAM complex expression and the differences in methylation between tumors and corresponding normal tissues. The Cox proportional hazard model was used to calculate survival risk, and H.R. The Kaplan-Meier survival curve was used to estimate the prognostic significance of each variable. The log-rank test was used for comparison. Unless otherwise specified, the rank-sum test detects two sets of data, and a *p*-value < 0.05 is considered statistically significant.

## Results

### Gene Set Expression and Subtype Analysis of the DREAM Complex

**FIGURE 1:**
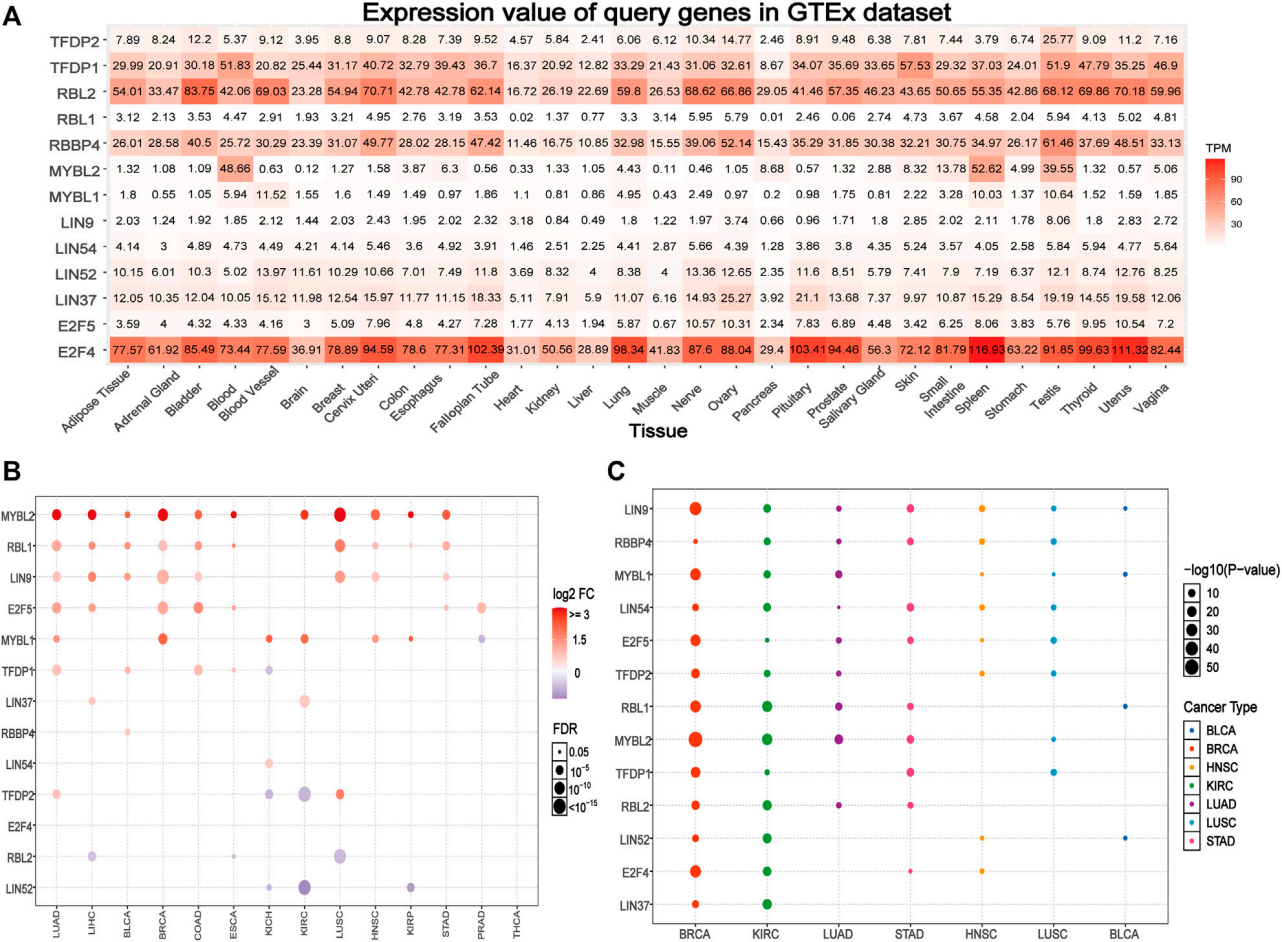
Gene expression of the DREAM complex. **(A)** Heatmap of the expression profiles of the DREAM complex in the GTEx dataset. **(B)** Differences in mRNA levels between normal samples and adjacent cancers. **(C)** The expression of subtypes of the DREAM complex in tumors.

Based on the GTEx data set, we explored the differential expression of the DREAM complex in normal tissues. As shown in Figure 1A, the expression of TFDP1 in the Blood, Skin, and Testis, the expression of RBL2 in Bladder, Cervix Uteri and the Uterus, the expression of RBBP4 in the Testis, the expression of MYBL2 in Blood, Spleen and the Testis, and the expression of E2F4 in Spleen, Uterus, and Pituitary were significantly upregulated. At the same time, we found that the DREAM complex was abnormally expressed in 14 solid tumors. (*p* < 0.05, Figure 1B). The expression levels of RBL2 and LIN52 were significantly downregulated in numerous cancers. The expression levels of MYBL2, RBL1, LIN9, and E2F5 in various cancers were significantly upregulated. However, there was no significant difference in the expression of the DREAM complex in THCA. In addition, to identify clinically relevant genes that affect tumor subtypes, we evaluated the expression of subtypes of the DREAM complex in cancers. We found that LIN9, MYBL1, and MYBL2 in BRCA; RBL1, MYBL2, and RBL2 in KIRC; RBL1, MYBL2 and RBL2 in LUAD; LIN9, LIN54, MYBL2, and TFDP1 in STAD; LIN9 and RBBP4 in HNSC; LIN9, RBBP4, and LIN54 in LUSC; LIN9 and MYBL1, RBL1 and LIN52 in BLCA were significantly differentially expressed (*p* < 0.05, Figure 1C). Expression survival analysis showed that MYBL2, which showed increased expression in KIRC, LGG, KICH, ACC, MESO, SARC, ESCA, KIRP, LIHC, and LUAD; MYBL1 in KIRC, LGG, KICH, ACC, MESO, KIRP, and PAAD; LIN9 in LGG, KICH, and ACC; LIN37 in KIRC and MESO; was related to poor survival. The genes with decreased expression, TFDP1, RBL2, LIN52, and LIN54 in KIRC; TFDP1 in LGG; RBL2 and E2F4 in KICH; RBL2 in ACC; RBBP4, LIN52 and E2F5 in GBM; RBL1 and TFDP2 in READ; and TFDP2 in UVM, were associated with poor survival (*p* < 0.05, Supplementary Figure S1A). We compared the DREAM complex transcription levels through the Oncomine database (https://www.oncomine.org/resource/login.html, an online cancer microarray database) in cancer and normal samples. Compared with the normal samples, the expression level of the DREAM complex mRNA in cancer was significantly upregulated (Supplementary Figure S1B). Survival analysis of six significantly differential genes showed that high expression of E2F4, LIN9, MYBL1, MYBL2, RBL1, and TFDP1 were associated with poor overall survival in the GEPIA2 database (*p* < 0.05, Supplementary Figure S1C). The above results indicated that the abnormal expression of the DREAM complex might was involved in the occurrence of tumors.

### Somatic Mutations of the DREAM Complex

**FIGURE 2:**
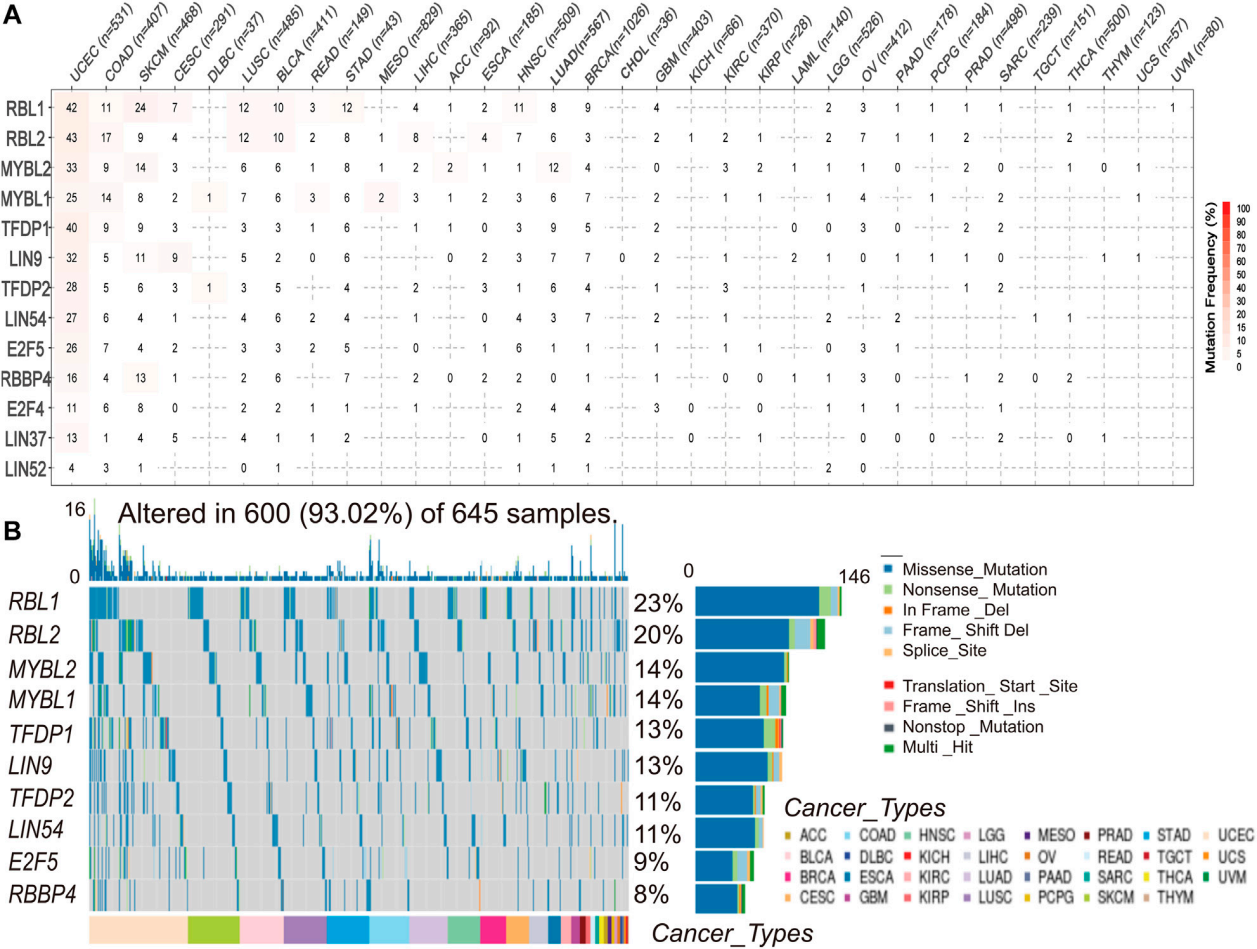
Single nucleotide variation (SNV) frequency and variant types of the DREAM complex. **(A)** Mutation frequency of the DREAM complex. The number indicates the number of samples with corresponding mutations in a specific cancer. “0” means that there is no mutation in the coding region of the gene, and no number indicates that there are no mutations in any region of the gene. **(B)** SNV oncoplot. The waterfall chart shows the mutation distribution and classification of SNV types of the DREAM complex.

To understand and compare the frequency and variant types of the DREAM complex in each cancer subtype, we analyzed data from The Cancer Genome Atlas Network (TCGA) studies. As shown in Figure 2A, SNV frequencies of UCEC, SKCM, and COAD were 1%–43% in these cancers. The SNV frequency of the DREAM complex was 93.02% (600 of 645 tumors). SNV analysis indicated that the main frequent variant classification of the DREAM complex gene mutation was missense mutation. SNV percentage analysis demonstrated that the top 10 mutated genes were RBL1, RBL2, MYBL2, MYBL1, TFDP1, LIN9, TFDP2, LIN54, E2F5, and RBBP4, of which the mutation percentages were 23%, 20%, 14%, 14%, 13%, 13%, 11%, 11%, 9%, and 8%, respectively. The SNV frequency of the DREAM complex was increased in UCEC, SKCM, KIRC, and LUSC (Figure 2B).

### Copy Number Variation of the DREAM Complex

**FIGURE 3:**
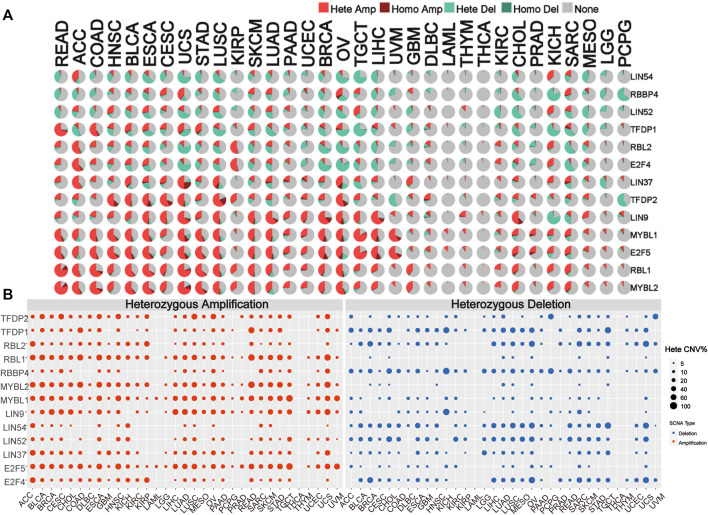
Copy number variation (CNV) is the basis of the DREAM complex dysregulation. **(A)**The copy number variation (CNV) pie chart shows the proportion of different types of copy number variation for each gene in different cancers. Hete Amp, heterozygous amplification; Hete Del, heterozygous deletion; Homo Amp, homozygous amplification; Homo Del, homozygous deletion; None, no CNV. **(B)** The heterozygous CNV profile shows the percentage of heterozygous CNV, including the percentage of heterozygous CNV amplification and deletion for each gene in each cancer. In each type of cancer, only genes with a CNV of 5% are shown as a dot in the graph.

To identify alteration in CNV, we analyzed the CNV data of the DREAM complex in the TCGA database. The CNV pie chart distribution showed that these genes’ main copy number variants include heterozygous amplification and deletion. CNV percentage analysis indicated that heterozygous amplification of LIN54 in ACC and KICH; RBBP4 in CESC, UCS, O.V. and SARC; LIN52 in HNSC, ESCA, TGCT and KICH; TFDP1 in READ, COAD, BLCA, UCS and STAD; RBL2 in ACC, KIRP and KICH; E2F4 in ACC, KIRP and KICH; LIN37 in ACC, BLCA, CESC,UCS, LUSC, O.V. and GBM; TFDP2 in HNSC, BLCA, ESCA, CESC, UCS, LUSC KIRP and O.V.; LIN9 in BLCA, ESCA, CESC, UCS, LUSC, SKCM, LUAD, PAAD, UCEC, BRCA, O.V., TGCT, LIHC and CHOL; MYBL1 in READ, ACC, COAD, HNSC, BLCA, ESCA, CESC, UCS, STAD, LUSC, SKCM, LUAD, UCEC, BRCA, O.V., TGCT, LIHC, and UVM; E2F5 in READ, ACC, COAD, HNSC, BLCA, ESCA, CESC, UCS, STAD, LUSC, SKCM, LUAD, UCEC, O.V., TGCT, LIHC, and UVM CHOL,SARC; RBL1 in READ, ACC, COAD, HNSC, BLCA, ESCA, CESC, UCS, STAD, LUSC, KIRP, SKCM, LUAD, BRCA, O.V., TGCT, LIHC,GBM, CHOL, KICH and SARC; MYBL2 in READ, ACC, COAD, HNSC, BLCA, ESCA, CESC, UCS, STAD, LUSC, KIRP, SKCM, LUAD, BRCA, O.V., TGCT, LIHC,GBM, CHOL, KICH and SARC was all more significant than 25% (*p* < 0.05, Figures 3A,B). Homozygous analysis showed that the amplified genes were TFDP2 in CESC, ESCA, HNSC, LUSC, and O.V.; TFDP1 in SARC; RBL1 in COAD and READ; RBBP4 in O.V.; MYBL2 in COAD, READ, STAD and UCS, MYBL1 in BRCA, LIHC, O.V., PRAD, UCS and UVM, LIN9 in BRCA, CHOL, LIHC, LUAD, O.V. and UCS, LIN37 in LUAD, LUSC, OV SARC, and UCS, and E2F5 in BLCA, BRCA, CHOL, ESCA, LIHC, O.V., PRAD, UCS and UVM (*p* < 0.05, Supplementary Figure S2A). In addition, we also found that mRNA expression is positively correlated with CNV, especially E2F4 and TFDP1 in BRCA, and RBBP4 in LGG (*p* < 0.05, Supplementary Figure S2B). These results indicated that the DREAM complex had heterozygous amplification and heterozygous deletion, which mediated its abnormal expression and might play an essential role in the occurrence and development of tumors.

### Methylation Analysis of DREAM Complex

**FIGURE 4:**
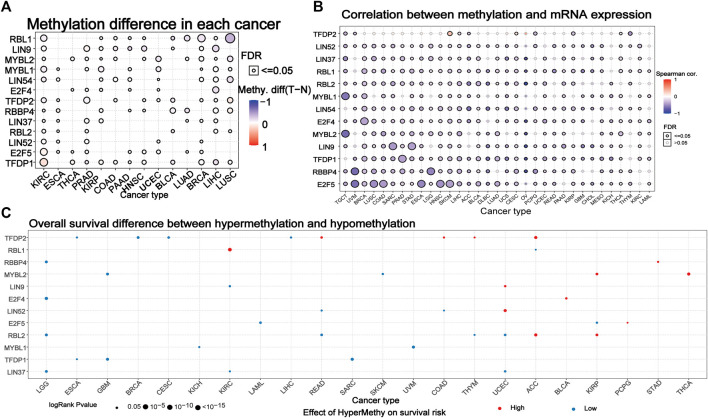
Methylation of the DREAM complex. **(A)** Differential methylation of the DREAM complex between 14 paired normal and tumor tissues. Blue dots indicate a decrease in the degree of methylation in the tumor, red dots indicate an increase in the degree of methylation in the tumor, the darker the color, the greater the difference in the degree of methylation. **(B)** Correlation between methylation and mRNA gene expression. Blue represents negative correlation, red represents positive correlation, the darker the color, the higher the correlation. **(C)** Survival difference between samples with the DREAM complex with high and low methylation. The red dot indicates that the survival rate of the hypermethylation group is lower, and the blue dot indicates that the survival rate of the hypomethylation group is better. The size of a point indicates statistical significance, and the larger the size of the point, the higher the statistical significance.

We explored the methylation of the DREAM complex to determine epigenetic regulation. The methylation of the DREAM complex in different tumors was highly heterogeneous. There were more hypermethylated genes than hypomethylated genes in KIRC, PRAD, KIRP, COAD, PAAD, HNSC, UCEC, LUAD, BRCA, LIHC, and LUHC. Whereas there were more hypomethylated than hypermethylated genes in BLCA. The MYBL2, MYBL1, LIN54, E2F4, RBBP4, LIN37, RBL2, LIN52, E2F5, and TFDP1 were hypermethylated in most cancers (*p* < 0.05). RBL1 was hypomethylated in LUSC, BRCA, LUAD, BLCA, and KIRC (*p* < 0.05). LIN9 was hypomethylated in LIHC, HNSC, PAAD, and COAD. TFDP2 was hypomethylated in KIRC, PRAD, and BRCA (Figure 4A). We then analyzed the correlation between methylation and mRNA expression, and the results showed that the expression level of most genes was negatively correlated with the methylation level. Only the methylation of TFDP2 in SKCM and CESC was positively associated with gene expression (*p* < 0.05, Figure 4B). Survival analysis showed that hypermethylation of TFDP2, RBL1, and MYBL2 was associated with poor survival in most cancers. In addition, the hypomethylation of RBL2, MYBL1, TFDP1, and LIN37 was mainly related to poor survival (*p* < 0.05, Figure 4C). The above analysis showed that abnormal DNA methylation may regulate the odd expression of the DREAM complex and was related to the prognosis of cancer patients.

### Pathway Activity Analysis

**FIGURE 5:**
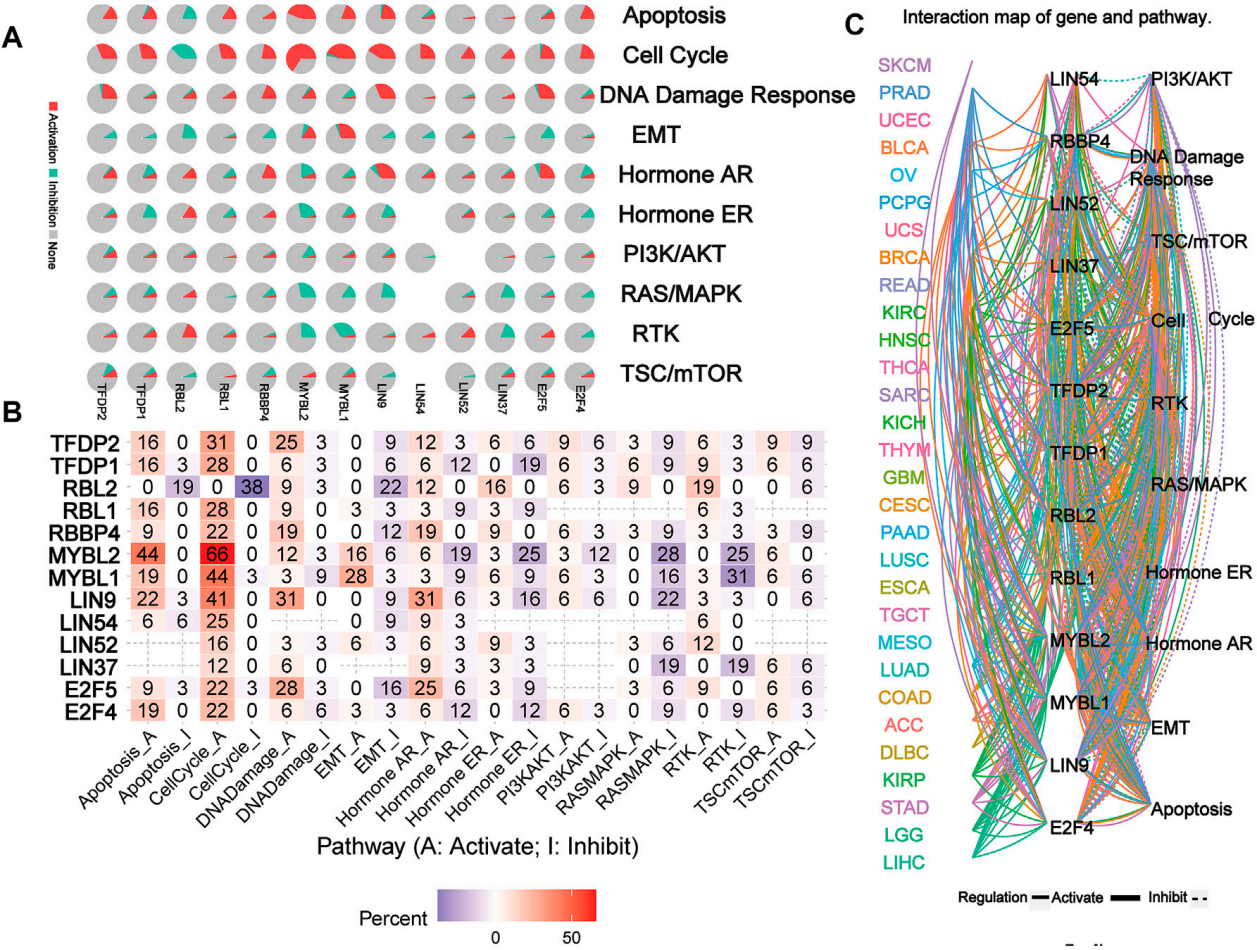
The pathway network between the DREAM complex. **(A)** The pie chart shows the global percentage of cancers where one gene affects the pathways of 32 cancers. **(B)** The combined percentage of the DREAM complex’s influence on pathway activity. **(C)** A network showing the relationship between genes and pathways is connected by straight lines. The solid line represents activation and the dashed line represents inhibition. The colors of the lines represent different types of cancer.

The network of related pathways showed that the DREAM complex was significantly involved in tumor-related signaling pathways, including TSC/mTOR, RTK, RAS/MAPK, PI3K/AKT, Hormone ER, Hormone AR, EMT, DNA damage response, cell cycle, and apoptosis pathways (Figure 5A). TFDP2 was mostly involved in the activation of Apoptosis (16% activation), cell cycle (31% activation) and DNA damage (25% activation); TFDP1 was mostly involved in the activation of Apoptosis (16% activation), cell cycle (28% activation) and the inactivation of Hormone ER (19% inhibition). However, for RBL2, the main activated pathways were Apoptosis (0% activation vs. 19% inhibition), cell cycle (0% activation vs. 38% inhibition), EMT (0% activation vs. 22% inhibition) and RTK (19% activation vs. 0% inhibition); for RBL1, the most activated pathways were Apoptosis (16% activation vs. 0% inhibition) and cell cycle (28% activation vs. 0% inhibition); for RBBP4, the main activated pathways were cell cycle (22% activation vs. 0% inhibition), DNA damage (19% activation vs. 0% inhibition) and Hormone AR (19% activation vs. 0% inhibition); and for MYBL2, the main activated pathway was Apoptosis (44% activation vs. 0% inhibition), cell cycle (66% activation vs. 0% inhibition), Hormone AR (6% activation vs. 19% inhibition), Hormone ER (3% activation vs. 25% inhibition), RASMAPK (0% activation vs. 28% inhibition) and RTK (0% activation vs. 25% inhibition); for MYBL1, the main activated pathway was Apoptosis (19% activation vs. 0% inhibition), cell cycle (44% activation vs. 3% inhibition), EMT (28% activation vs. 3% inhibition), RASMAPK (0% activation vs. 16% inhibition) and RTK (3% activation vs. 31% inhibition); for LIN9, the main activated pathway was Apoptosis (22% activation vs. 3% inhibition), cell cycle (41% activation vs. 0% inhibition), DNA damage (31% activation vs. 0% inhibition) and Hormone AR (31% activation vs. 6% inhibition); for LIN54 and LIN52, the main activated pathway was cell cycle (25% activation vs. 0% inhibition and 16% activation vs. 0% inhibition); for LIN37, the main activated pathway was RASMAPK (0% activation vs. 19% inhibition) and RTK (0% activation vs. 19% inhibition); for E2F5, the main activated pathway was cell cycle (22% activation vs. 3% inhibition), DNA damage (28% activation vs. 3% inhibition) and Hormone AR (25% activation vs. 6% inhibition); for E2F5, the main activated pathway was Apoptosis (19% activation vs. 0% inhibition), cell cycle (22% activation vs. 0% inhibition) (*p* < 0.05, Figure 5B). However, network analysis showed that LIN54 did not participate in the PI3K/AKT pathway of LUAD; RBBP4 did not participate in the TSC/mTOR and PI3K/AKT pathways of THCA (*p* < 0.05, Figure 5C). These results indicated that the DREAM complex plays a crucial role in regulating cancer-related pathways.

### Drug Sensitivity Analysis

**FIGURE 6:**
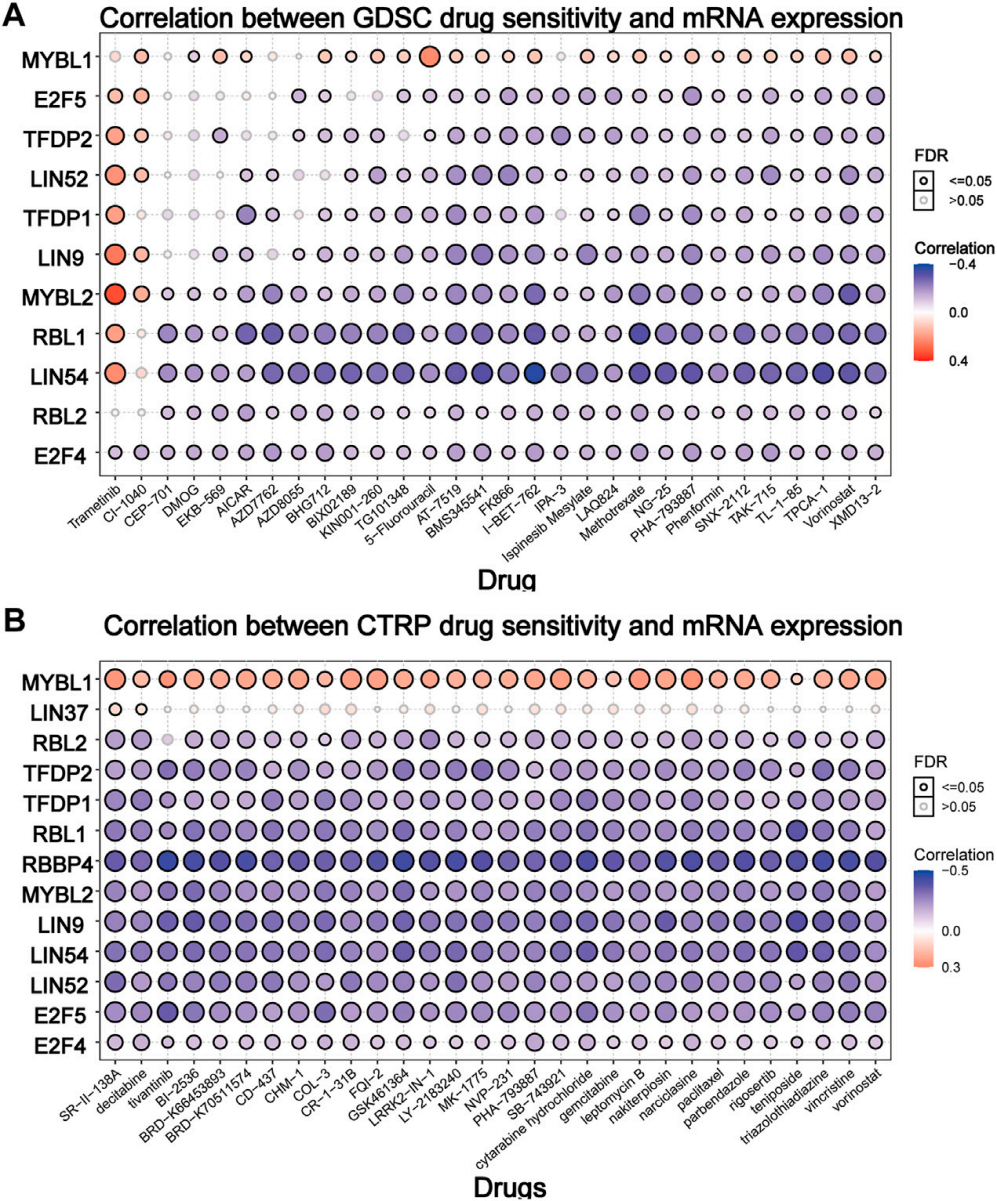
Drug sensitivity analysis of the DREAM complex. The gene set drug resistance analysis from Genomics of Drug Sensitivity in Cancer (GDSC) IC50 drug data and The Cancer Therapeutics Response Portal (CTRP). Spearman’s correlation represents how the gene expression correlates with a drug. The horizontal axis represents different drugs. Blue represents negative correlations and red represents positive correlations. Black circles indicate FDR < 0.05 and gray circles indicate FDR > 0.05.

To elucidate the effect of the DREAM complex on the therapeutic effect of chemotherapeutics, we integrated the drug sensitivity and gene expression profile data of tumor cell lines provided by GDSC and CTRP. Spearman’s correlation analysis showed that drug sensitivity toward DMOG (Dimethyloxalylglcine) correlated with the expression of MYBL1 (negative correlation with IC50). However, drug resistance toward Trametinib, CI-1040 associated with the expression of E2F5, TFDP2, LIN52, LIN9, and MYBL2 (positive correlation with IC50) (*p* < 0.05, Figure 6A). Drug resistance toward all drugs in Figure 6B correlated with the expression of MYBL1 (positive correlation with IC50). Drug sensitivity toward all drugs in Figure 6B correlated with the expression of RBL2, TFDP2, TFDP1, RBL1, RBBP4, MYBL2, LIN9, LIN54, LIN52, E2F5, and E2F4 (negative correlation with IC50). These results indicated that the abnormal expression of the DREAM complex might mediate resistance to chemotherapy and targeted drug therapy.

## Discussion

In the cell cycle process, the coordinated expression of periodic related genes can maintain the integrity of the genome and ensure cell proliferation so as not to be blocked by environmental signals. The DREAM complex, also known as the LINC complex, is an evolutionary conserved cell cycle regulatory protein complex. More and more evidence shows that the DREAM complex has a potential inhibitory effect in tumorigenesis (Ho et al., 2009; Kim et al., 2021). In different organisms, the destruction of the DREAM complex can lead to developmental defects, genomic instability, tumorigenesis, and increased mortality (Malumbres and Barbacid, 2009; Reichert et al., 2010; Hauser et al., 2012). Therefore, studying the related mechanisms of the DREAM complex in cancer is necessary to understand tumorigenesis and explore potential targets for clinical treatment. We have performed a comprehensive and systematic characterization of the DREAM complex in multiple samples of 33 cancers by mining multiple sets of analysis data. Our research results reveal a variety of potential mechanisms of the DREAM complex in the cancer environment and reveal common signal pathways related to cancer pathways, thereby elucidating the overall regulation of the DREAM complex in cancer.

Our genetic analysis shows that the copy number of the DREAM complex gene changes frequently. We also found that mRNA expression is positively related to CNV, especially E2F4 and TFDP1 in BRCA and RBBP4 in LGG. The expression analysis of the DREAM complex confirms that the change of copy number is positively correlated with the expression, indicating that the shift in copy number can affect the expression of clock genes, thereby promoting the occurrence of tumors. Recent studies have also shown that MYBL2 is significantly upregulated in endometrial cancer (E.C.) and esophageal squamous cell carcinoma (ESCC), accompanied by significant copy number changes (CNA). Copy number amplification strengthens the expression of MYBL2 mRNA, leading to poor prognosis and severe E.C. pathology (Qin et al., 2016; Le et al., 2021).

Epigenetic analysis showed that the methylation of the DREAM complex in different tumors is highly heterogeneous. The MYBL2, MYBL1, LIN54, E2F4, RBBP4, LIN37, RBL2, LIN52, E2F5, and TFDP1 were hypermethylated in most cancers. At the same time, RBL1 was hypomethylated in LUSC, BRCA, LUAD, BLCA, and KIRC. The correlation between methylation and mRNA expression showed that the expression level of most genes was negatively correlated with the methylation level. Meanwhile, survival analysis showed that hypermethylation of TFDP2, RBL1, and MYBL2 was associated with poor survival in most cancers. A new DNA methylation 10-CPG disease-free survival prognosis shows that MYBL2 is associated with a high risk of prostate cancer (Hou et al., 2020). Homomorphic diffuse gliomas occur in children and adults and have concise morphologies with frequent MYBL1 and MYB changes and specific DNA methylation features (Wefers et al., 2020). Therefore, we speculated that the genetic and epigenetic modifications of the DREAM complex might promote tumors in some cases.

The DREAM complex gene inhibits cell apoptosis, cell cycle, and DNA damage response, which have been confirmed in several cancers (Musa et al., 2017; Iness and Litovchick, 2018; Bayley et al., 2020; Kim et al., 2021; Liu et al., 2021). These results indicate that the DREAM complex plays a crucial role in regulating cancer-related pathways and is consistent with our analysis results. In addition, our results show that MYBL2 and MYBL1 genes are also involved in the activation of the EMT pathway to promote tumor metastasis, which has also been confirmed in some tumors (Togashi et al., 2018; Xie et al., 2020; Li et al., 2021; Sakuma et al., 2021).

These results indicate that the DREAM complex constitutes an interactive network of tumor-related signaling pathways and may be involved in inhibiting various tumor progression and improving survival rates. Our drug sensitivity analysis showed that drug resistance toward Trametinib, CI-1040 was associated with the expression of E2F5, TFDP2, LIN52, LIN9, and MYBL2. However, these drugs’ potential mechanism of action on the DREAM complex expression and tumor progression remains to be further studied.

In summary, we have elucidated the genomics and clinical characteristics of the DREAM complex in 33 solid tumors. Our research found that the ectopic expression of the DREAM complex mediated by genome changes is involved in the activation of cancer-related pathways. Targeting the DREAM complex may be a novel and meaningful way to treat cancer. Our findings are significant and provide new insights into the regulation of the DREAM complex in tumors. The DREAM complex has various regulatory levels such as genetic and epigenetic changes, mRNA expression, and pathway correlation. These differences may lead to differences in drug efficacy, treatment response, and patient survival. Although our research provides new insights into the changes in the DREAM complex regulation, this analysis still has some limitations. First of all, based on the information of TCGA, we can only assess the overall genetic changes of tumor tissues and cannot analyze the genetic differences of tumor cells. Second, we only explored some potential changes that may affect the expression and function of the DREAM complex. Post-transcriptional and post-translational modifications, such as changes in mRNA splicing, m6A methylation, and protein stability, still require further consideration. Third, since our research is mainly based on gene expression, we cannot explore the role of CNV and SNV changes in tumors. Therefore, future research to comprehensively investigate cancer heterogeneity and individualized treatment are necessary.

## Data Availability

The data comes from the following sources available in the public domain: The Genotype-Tissue Expression (GTEx) dataset (V7.0) (https://commonfund.nih.gov/GTEx/); The Cancer Genome Atlas (TCGA) (https://portal.gdc.cancer.gov/); The cancer proteome atlas (TCPA) (https://tcpap ortal.org/tcpa/index.html); The GSCALite (http://bioinfo.life.hust.edu.cn/web/GSCALite/) and The Genomics of Drug Sensitivity in Cancer (GDSC) database (www.cance rrxge ne.org).

## Supplementary Material

Supplementary Figure S1 Survival analysis of the DREAM complex. **(A)** The size of the dot represents the importance of the gene’s impact on the survival of each cancer, and the *p* value is obtained by Kaplan-Meier analysis. A red dot indicates that the survival rate expressed in the cancer type is poor. The blue dot indicates that the survival rate expressed in the cancer type is better. **(B)** The DREAM complex family members in different types of cancers (ONCOMINE). The figure shows the number of data sets with target gene mRNA over-expression (red) or downregulated (blue). **(C)** The expression of E2F4, LIN9, MYBL1, MYBL2, RBL1 and TFDP1 were associated with poor overall survival in the GEPIA2 database.

Supplementary Figure S2 CNV correlation with mRNA expression. **(A)** The homozygous CNV profile shows the percentage of homozygous CNV. **(B)** The association between paired mRNA expression and CNV percentage in samples was based on a Person’s product moment correlation coefficient. The size of the point represents the statistical significance, where the bigger the dot size, the higher the statistical significance. FDR, false discovery rate.

## References

[B1] Akbani R. Ng P. K. S. Werner H. M. J. Shahmoradgoli M. Zhang F. Ju Z. (2014). A Pan-Cancer Proteomic Perspective on the Cancer Genome Atlas. Nat. Commun. 5, 3887. 10.1038/ncomms4887 24871328 PMC4109726

[B2] Basu A. Bodycombe N. E. Cheah J. H. Price E. V. Liu K. Schaefer G. I. (2013). An Interactive Resource to Identify Cancer Genetic and Lineage Dependencies Targeted by Small Molecules. Cell 154 (5), 1151–1161. 10.1016/j.cell.2013.08.003 23993102 PMC3954635

[B3] Bayley R. Ward C. Garcia P. (2020). MYBL2 Amplification in Breast Cancer: Molecular Mechanisms and Therapeutic Potential. Biochim. Biophys. Acta (Bba) - Rev. Cancer 1874 (2), 188407. 10.1016/j.bbcan.2020.188407 32853735

[B4] Fischer M. Grossmann P. Padi M. DeCaprio J. A. (2016). Integration of TP53, DREAM, MMB-FOXM1 and RB-E2f Target Gene Analyses Identifies Cell Cycle Gene Regulatory Networks. Nucleic Acids Res. 44 (13), 6070–6086. 10.1093/nar/gkw523 27280975 PMC4994865

[B5] Gal C. Carelli F. N. Appert A. Cerrato C. Huang N. Dong Y. (2021). DREAM Represses Distinct Targets by Cooperating with Different THAP Domain Proteins. Cel Rep. 37 (3), 109835. 10.1016/j.celrep.2021.109835 PMC8552245 34686342

[B6] Harrison M. M. Ceol C. J. Lu X. Horvitz H. R. (2006). Some C. elegans Class B Synthetic Multivulva Proteins Encode a Conserved LIN-35 Rb-Containing Complex Distinct from a NuRD-like Complex. Proc. Natl. Acad. Sci. U.S.A. 103 (45), 16782–16787. 10.1073/pnas.0608461103 17075059 PMC1636532

[B7] Hauser S. Ulrich T. Wurster S. Schmitt K. Reichert N. Gaubatz S. (2012). Loss of LIN9, a Member of the DREAM Complex, Cooperates with SV40 Large T Antigen to Induce Genomic Instability and anchorage-independent Growth. Oncogene 31 (14), 1859–1868. 10.1038/onc.2011.364 21860417

[B8] Ho V. M. Schaffer B. E. Karnezis A. N. Park K. S. Sage J. (2009). The Retinoblastoma Gene Rb and its Family Member P130 Suppress Lung Adenocarcinoma Induced by Oncogenic K-Ras. Oncogene 28 (10), 1393–1399. 10.1038/onc.2008.491 19151761 PMC2834234

[B9] Hou X. Zhang Y. Han S. Hou B. (2020). A Novel DNA Methylation 10-CpG Prognostic Signature of Disease-free Survival Reveal that MYBL2 Is Associated with High Risk in Prostate Cancer. Expert Rev. Anticancer Ther. 20 (12), 1107–1119. 10.1080/14737140.2020.1838280 33073649

[B10] Iness A. N. Litovchick L. (2018). MuvB: A Key to Cell Cycle Control in Ovarian Cancer. Front. Oncol. 8, 223. 10.3389/fonc.2018.00223 29942794 PMC6004728

[B11] Kim M. J. Cervantes C. Jung Y.-S. Zhang X. Zhang J. Lee S. H. (2021). PAF Remodels the DREAM Complex to Bypass Cell Quiescence and Promote Lung Tumorigenesis. Mol. Cel 81 (8), 1698–1714. 10.1016/j.molcel.2021.02.001 PMC8052288 33626321

[B12] Korenjak M. Taylor-Harding B. Binné U. K. Satterlee J. S. Stevaux O. Aasland R. (2004). Native E2F/RBF Complexes Contain Myb-Interacting Proteins and Repress Transcription of Developmentally Controlled E2F Target Genes. Cell 119 (2), 181–193. 10.1016/j.cell.2004.09.034 15479636

[B13] Le L. Luo J. Wu H. Chen L. Tang X. Fu F. (2021). Overexpression of MYBL2 Predicts Poor Prognosis and Promotes Oncogenesis in Endometrial Carcinoma. Eur. J. Histochem. 65 (2), 3226. 10.4081/ejh.2021.3226 PMC8054569 33782625

[B14] Lewis P. W. Beall E. L. Fleischer T. C. Georlette D. Link A. J. Botchan M. R. (2004). Identification of a Drosophila Myb-E2f2/RBF Transcriptional Repressor Complex. Genes Dev. 18 (23), 2929–2940. 10.1101/gad.1255204 15545624 PMC534653

[B15] Li Q. Wang M. Hu Y. Zhao E. Li J. Ren L. (2021). MYBL2 Disrupts the Hippo-YAP Pathway and Confers Castration Resistance and Metastatic Potential in Prostate Cancer. Theranostics 11 (12), 5794–5812. 10.7150/thno.56604 33897882 PMC8058714

[B16] Litovchick L. Sadasivam S. Florens L. Zhu X. Swanson S. K. Velmurugan S. (2007). Evolutionarily Conserved Multisubunit RBL2/p130 and E2F4 Protein Complex Represses Human Cell Cycle-dependent Genes in Quiescence. Mol. Cel 26 (4), 539–551. 10.1016/j.molcel.2007.04.015 17531812

[B17] Liu C.-J. Hu F.-F. Xia M.-X. Han L. Zhang Q. Guo A.-Y. (2018). GSCALite: a Web Server for Gene Set Cancer Analysis. Bioinformatics 34 (21), 3771–3772. 10.1093/bioinformatics/bty411 29790900

[B18] Liu J. Xia L. Wang S. Cai X. Wu X. Zou C. (2021). E2F4 Promotes the Proliferation of Hepatocellular Carcinoma Cells through Upregulation of CDCA3. J. Cancer 12 (17), 5173–5180. 10.7150/jca.53708 34335934 PMC8317516

[B19] MacDonald J. Ramos-Valdes Y. Perampalam P. Litovchick L. DiMattia G. E. Dick F. A. (2017). A Systematic Analysis of Negative Growth Control Implicates the DREAM Complex in Cancer Cell Dormancy. Mol. Cancer Res. 15 (4), 371–381. 10.1158/1541-7786.mcr-16-0323-t 28031411

[B20] Malumbres M. Barbacid M. (2009). Cell Cycle, CDKs and Cancer: a Changing Paradigm. Nat. Rev. Cancer 9 (3), 153–166. 10.1038/nrc2602 19238148

[B21] Mayakonda A. Lin D.-C. Assenov Y. Plass C. Koeffler H. P. (2018). Maftools: Efficient and Comprehensive Analysis of Somatic Variants in Cancer. Genome Res. 28 (11), 1747–1756. 10.1101/gr.239244.118 30341162 PMC6211645

[B22] Mermel C. H. Schumacher S. E. Hill B. Meyerson M. L. Beroukhim R. Getz G. (2011). GISTIC2.0 Facilitates Sensitive and Confident Localization of the Targets of Focal Somatic Copy-Number Alteration in Human Cancers. Genome Biol. 12 (4), R41. 10.1186/gb-2011-12-4-r41 21527027 PMC3218867

[B23] Musa J. Aynaud M.-M. Mirabeau O. Delattre O. Grünewald T. G. (2017). MYBL2 (B-Myb): a central Regulator of Cell Proliferation, Cell Survival and Differentiation Involved in Tumorigenesis. Cell Death Dis 8 (6), e2895. 10.1038/cddis.2017.244 28640249 PMC5520903

[B24] Nguyen Q.-H. Le D.-H. (2020). Improving Existing Analysis Pipeline to Identify and Analyze Cancer Driver Genes Using Multi-Omics Data. Sci. Rep. 10 (1), 20521. 10.1038/s41598-020-77318-1 33239644 PMC7688645

[B25] Pilkinton M. Sandoval R. Colamonici O. R. (2007). Mammalian Mip/LIN-9 Interacts with Either the P107, p130/E2F4 Repressor Complex or B-Myb in a Cell Cycle-phase-dependent Context Distinct from the Drosophila dREAM Complex. Oncogene 26 (54), 7535–7543. 10.1038/sj.onc.1210562 17563750

[B26] Qin H.-D. Liao X.-Y. Chen Y.-B. Huang S.-Y. Xue W.-Q. Li F.-F. (2016). Genomic Characterization of Esophageal Squamous Cell Carcinoma Reveals Critical Genes Underlying Tumorigenesis and Poor Prognosis. Am. J. Hum. Genet. 98 (4), 709–727. 10.1016/j.ajhg.2016.02.021 27058444 PMC4833434

[B27] Rees M. G. Seashore-Ludlow B. Cheah J. H. Adams D. J. Price E. V. Gill S. (2016). Correlating Chemical Sensitivity and Basal Gene Expression Reveals Mechanism of Action. Nat. Chem. Biol. 12 (2), 109–116. 10.1038/nchembio.1986 26656090 PMC4718762

[B28] Reichert N. Wurster S. Ulrich T. Schmitt K. Hauser S. Probst L. (2010). Lin9, a Subunit of the Mammalian DREAM Complex, Is Essential for Embryonic Development, for Survival of Adult Mice, and for Tumor Suppression. Mol. Cel Biol 30 (12), 2896–2908. 10.1128/mcb.00028-10 PMC2876665 20404087

[B29] Sadasivam S. DeCaprio J. A. (2013). The DREAM Complex: Master Coordinator of Cell Cycle-dependent Gene Expression. Nat. Rev. Cancer 13 (8), 585–595. 10.1038/nrc3556 23842645 PMC3986830

[B30] Sadasivam S. Duan S. DeCaprio J. A. (2012). The MuvB Complex Sequentially Recruits B-Myb and FoxM1 to Promote Mitotic Gene Expression. Genes Dev. 26 (5), 474–489. 10.1101/gad.181933.111 22391450 PMC3305985

[B31] Sakuma K. Sasaki E. Hosoda W. Komori K. Shimizu Y. Yatabe Y. (2021). MYB Mediates Downregulation of the Colorectal Cancer Metastasis Suppressor Heterogeneous Nuclear Ribonucleoprotein L‐like during Epithelial‐mesenchymal Transition. Cancer Sci. 112 (9), 3846–3855. 10.1111/cas.15069 34286904 PMC8409424

[B32] Schlattl A. Anders S. Waszak S. M. Huber W. Korbel J. O. (2011). Relating CNVs to Transcriptome Data at fine Resolution: Assessment of the Effect of Variant Size, Type, and Overlap with Functional Regions. Genome Res. 21 (12), 2004–2013. 10.1101/gr.122614.111 21862627 PMC3227091

[B33] Schmit F. Korenjak M. Mannefeld M. Schmitt K. Franke C. von Eyss B. (2007). LINC, a Human Complex that Is Related to pRB-Containing Complexes in Invertebrates Regulates the Expression of G2/M Genes. Cell Cycle 6 (15), 1903–1913. 10.4161/cc.6.15.4512 17671431

[B34] Seashore-Ludlow B. Rees M. G. Cheah J. H. Cokol M. Price E. V. Coletti M. E. (2015). Harnessing Connectivity in a Large-Scale Small-Molecule Sensitivity Dataset. Cancer Discov. 5 (11), 1210–1223. 10.1158/2159-8290.cd-15-0235 26482930 PMC4631646

[B35] Togashi Y. Dobashi A. Sakata S. Sato Y. Baba S. Seto A. (2018). MYB and MYBL1 in Adenoid Cystic Carcinoma: Diversity in the Mode of Genomic Rearrangement and Transcripts. Mod. Pathol. 31 (6), 934–946. 10.1038/s41379-018-0008-8 29410490

[B36] Walston H. Iness A. N. Litovchick L. (2021). DREAM on: Cell Cycle Control in Development and Disease. Annu. Rev. Genet. 55, 309–329. 10.1146/annurev-genet-071819-103836 34496610

[B37] Wefers A. K. Stichel D. Schrimpf D. Coras R. Pages M. Tauziède-Espariat A. (2020). Isomorphic Diffuse Glioma Is a Morphologically and Molecularly Distinct Tumour Entity with Recurrent Gene Fusions of MYBL1 or MYB and a Benign Disease Course. Acta Neuropathol. 139 (1), 193–209. 10.1007/s00401-019-02078-w 31563982 PMC7477753

[B38] Xie B. Liu Y. Zhao Z. Liu Q. Wang X. Xie Y. (2020). MYB Proto-oncogene-like 1-TWIST1 Axis Promotes Growth and Metastasis of Hepatocellular Carcinoma Cells. Mol. Ther. - Oncolytics 18, 58–69. 10.1016/j.omto.2020.05.016 32637581 PMC7327431

[B39] Ye Y. Xiang Y. Ozguc F. M. Kim Y. Liu C.-J. Park P. K. (2018). The Genomic Landscape and Pharmacogenomic Interactions of Clock Genes in Cancer Chronotherapy. Cel Syst. 6 (3), 314–328. 10.1016/j.cels.2018.01.013 PMC6056007 29525205

